# Enhanced Actuation Performance of Polymeric Composites by Simultaneously Incorporating Covalent-Bond-Functionalized Dielectric Nanoparticles and Polar Plasticizer

**DOI:** 10.3390/polym14194218

**Published:** 2022-10-08

**Authors:** Huiwan Lu, Dan Yang

**Affiliations:** 1 College of Materials Science and Engineering, Beijing University of Chemical Technology, Beijing 100029, China; 2 College of New Materials and Chemical Engineering, Beijing Institute of Petrochemical Technology, Beijing 102617, China

**Keywords:** dielectric elastomer, plasticizer, elastic modulus, actuated strain, silane

## Abstract

Dielectric elastomer actuators (DEAs), similar to artificial muscles, are widely applied in the fields of robotics and biomedical devices. In this work, 3-mercaptopropyl ethyoxyl di(tridecyl-pentaethoxy) silane (Si747)-modified BaTiO_3_ (BTO) nanoparticles (denoted as Si747@BTO) were utilized as dielectric filler to improve the dielectric constant while epoxy soybean oil (ESO) was employed as a plasticizer to decrease the elastic modulus, with the aim of improving the actuation performance of epoxy natural rubber (ENR) composites. The participation of Si747 in the vulcanization reaction of ENR led to the formation of covalent bonds between BTO and ENR chains, resulting in a uniform dispersion of BTO nanoparticles in the ENR matrix. Among obtained composites, the 50 phr ESO/Si747@BTO/ENR exhibited a relatively high actuated strain of 8.89% at 22 kV/mm, which is a value about 5.1-fold higher than that of pure ENR (1.45%) under the same electric field.

## 1. Introduction

Dielectric elastomer actuators (DEAs) when combined with dielectric elastomer (DE) films and compliant electrodes are useful for their large actuated strain, fast response, and lightweight properties [1,2,3]. When the two compliant electrodes of DEA are subjected to electric fields, the DE films would squeeze in the thickness direction and expand along the in-plane direction [4,5,6]. Similar to artificial muscles, DEAs have widely been applied in robotics, artificial muscles, sensors, wearable devices, and optical devices [7,8]. However, DEAs require high applied driving voltages, which are dangerous for the human body and equipment [9]. Moreover, the high operating voltages increase the construction cost and appearance size of the voltage source [10]. Therefore, preparing DEAs with large actuated strains at low driving voltages is highly desirable.

A large electromechanical sensitivity (*β*) (defined by the ratio between the dielectric constant (*ε_r_*) and Young’s modulus (*Y*)) could decrease the driving voltage of DEAs [11,12,13]. Usually, improving *ε_r_* and decreasing *Y* in DEs achieves larger *β* values [14]. Previous studies suggested that adding conductive fillers (such as silver (Ag), carbon nanotubes (CNTs), graphene oxide, and polyaniline) or high-*ε_r_* ceramic particles (such as barium titanate (BTO), calcium copper titanate (CCTO), and titanium dioxide (TiO_2_)) into polymeric matrices may improve the *ε_r_* of polymeric composites [15,16]. Huang et al. [17] prepared high-*ε_r_* polytetrafluoroethylene/carbon nanotube (PTFE/CNT) nanofiber membranes via the electrostatic spinning method and high-temperature sintering. The *ε_r_* of the resulting PTFE/CNT nanofiber membrane reached 58.46 at 100 Hz when filled with 5 wt% CNTs. However, the dielectric loss tangent (*tanδ*) reached 2372.3 at 100 Hz, while the conductivity increased to 1.5 S/m; however, such features are not conducive to DE as insulating capacitors.

The incorporation of ceramic fillers, such as BTO, into polymeric matrices would not only increase the *ε_r_*, but also maintain low *tanδ* [18,19]. However, ceramic nanoparticles often agglomerate with each other due to their high surface energies [20]. Consequently, modifying the surface of ceramic nanoparticles to ensure uniform dispersion in polymeric matrices is of great importance [21,22]. Jiang et al. [11] used walnut polyphenols to modify BTO particles, followed by filling them into a silicone rubber (SR) matrix to yield DE composites. Under the applied electric field of 187 V/μm, the maximum actuated strain of the resulting SR/WNBT composites reached 38% with 5 wt% WNBT particles. However, the non-covalent bond modification usually induced a weak interface between the filler and the matrix. By contrast, the covalent bond modification could provide strong interface interactions between the dielectric nanoparticles and rubber chains, which is beneficial for improving the durability of DEAs [23,24]. The coupling agent 3-mercaptopropyl ethyoxyl di(tridecyl-pentaethyoxyl) silane (Si747), containing hydrosulphonyl (-SH) groups, could participate in the vulcanization of rubber, thereby providing strong interface interactions between the dielectric nanoparticles and rubber chains [23].

Furthermore, reducing the *Y* of DE composites by adding plasticizers could increase *β* [25]. Nie et al. [26] used dibutyl phthalate (DBP) as a plasticizer to enhance the electromechanical performance of fluorine-rich poly(vinylidene fluoride-co-hexafluoropropylene) P(VDF-HFP). At 25 MV/m, the bending angle of the composite filled with 10 wt% DBP reached 24.8°, a value that is 40% higher than that of pure P(VDF-HFP) (17.7°). Moreover, epoxy soybean oil (ESO) has been used as a polar plasticizer to yield lower *Y* DE composites [27,28].

Epoxy natural rubber (ENR) is a good candidate for DEs due to its good air tightness, damping, oil resistance, and skid resistance. More importantly, large amounts of polar epoxy groups in ENR chains may induce high dielectric constants (8.2 at 100 Hz) [29]. The vulcanization system is also indispensable for preparing rubbers; as such, this may not only improve the vulcanization rate and efficiency of rubber, but also improve its cross-linking density, which is conducive to preparing cured-elastomer composites with excellent performance [30].

In this work, the Si747-modified BTO nanoparticles (denoted as Si747@BTO) were used as high-*ε_r_* fillers, while the ESO was employed as a plasticizer for improving the actuation performance of ENR composites. The resulting *β* values were largely enhanced by simultaneously increasing *ε_r_* and decreasing *Y*. Among the obtained composites, 50 phr ESO/Si747@BTO/ENR showed a maximum actuated strain of 8.89% at a relatively low electric field of 22 kV/mm, which is a value about 5.1-fold higher than that of pure ENR (1.45%) under the same electric field.

## 2. Experimental Methods

### 2.1. Raw Materials

The BTO nanoparticles (at an average diameter of 100 nm) were supplied by Sinopharm Chemical Reagent Co. Ltd., Beijing, China. Tris(hydroxymethyl) aminomethane (Tris) and Si747 were obtained from Beijing Hwrkchemical Co., Ltd., Beijing, China. The commercial grade ENR was received from Shandong Li’ang New Material Technology Co., Ltd., Shandong, China. ESO, with an epoxy value of 6.0, was provided by Beijing Yinuokai Technology Co., Ltd., Beijing, China. The other chemicals were purchased from Sigma-Aldrich Co., Ltd., Shanghai, China.

### 2.2. Preparation of Si747@BTO Nanoparticles

**Figure 1:**
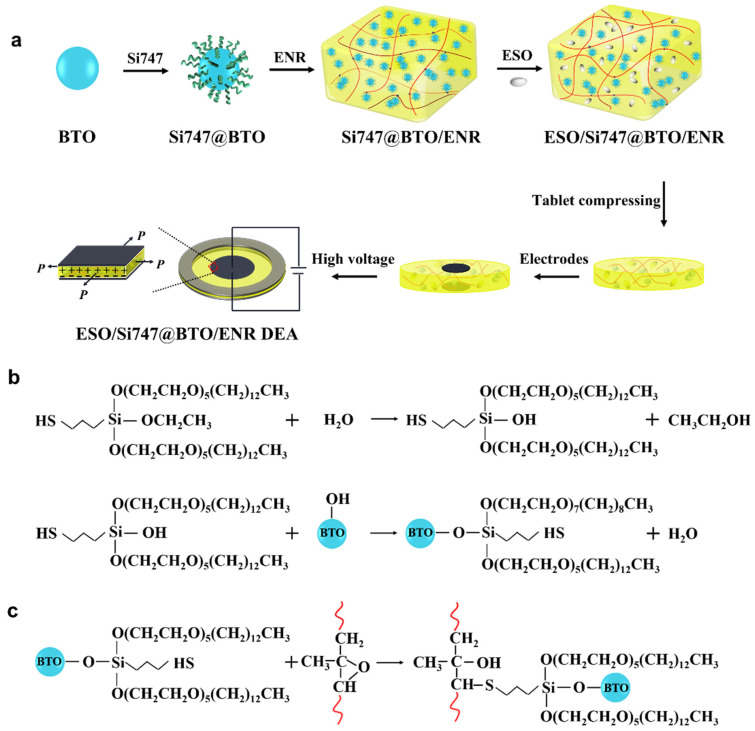
(**a**) Preparation and actuation mechanism of ESO/Si747@BTO/ENR DEA. (**b**) Reaction mechanism between Si747 and BTO nanoparticles. (**c**) Mechanism of Si747 reaction with ENR chains.

The detailed preparation process of Si747@BTO nanoparticles and ENR composites is shown in Figure 1a. First, 4 g of BTO nanoparticles was dispersed in 100 mL-distilled water at room temperature under ultrasonic treatment for 30 min; the pH of the dispersion was adjusted to 11 by adding Tris. Second, 0.2 g of Si747 was added dropwise into the above mixture alongside continuous stirring for 6 h at 60 °C. Finally, Si747@BTO was obtained by reflux extraction, vacuum filtration, and drying in a vacuum-drying oven at 60 °C for 24 h.

### 2.3. Preparation of ENR DE Composites

The ENR compounds were finished on a two-roll mill. According to the formula shown in experiment 3.1, different amounts of BTO or Si747@BTO nanoparticles (10, 20, and 30 phr (g per 100 g)); 1.5 g of sulfur (S); 5 g of zinc oxide (ZnO); 1 g of stearic acid; 0.5 g of dibenzothiazole disulfide (DM); and 1.5 g of n-cyclohexyl-2-benzothiazolides sulfonamide (CZ) were dispersed in 100 g ENR to obtain Si747@BTO/ENR compounds. According to the formula shown in experiment 3.2, different weight contents of ESO (10, 30, and 50 phr); 10 g of Si747@BTO; 1.5 g of S; 5 g of ZnO; 1 g of stearic acid; 0.5 g of DM; and 1.5 g of CZ were dispersed in 100 g of ENR to yield ESO/Si747@BTO/ENR compounds. The as-obtained compounds were cured by hot pressing (pressure: 25 MPa, temperature: 150 °C) at the optimal curing time (Tc 90) to yield cured ENR composites. The reference materials, i.e., the BTO/ENR composites, were also prepared according to the same procedure.

### 2.4. Characterization

The thermal gravimetric analysis (TGA) of BTO and Si747@BTO nanoparticles was performed on a TA SDT650 instrument under an N_2_ atmosphere (20 mL/min) from room temperature to 800 °C at a heating rate of 10 °C/min. A platinum crucible was used for the heating. Fourier transform infrared (FTIR) spectroscopy was carried out on an FTIR spectrometer Nicolet 6700. The chemical compositions of BTO nanoparticles, before and after modification, were identified by X-ray photoelectron spectroscopy (XPS, ESCALAB 250, Thermo Electron Corporation, USA) with Al Kα monochromated energy (150 W, 20 eV pass energy, and 500 µm spot size). The binding energies (BE) were calibrated using the C1s level at 284.8 eV as an internal standard. The micro-morphologies of ENR composites were analyzed via scanning electron microscopy (SEM, Hitachi S4800, Japan) at an accelerating voltage of 20 kV. The samples used for SEM testing were first broken by liquid nitrogen. A layer of gold was then sprayed on the fracture surface before testing. The stress–strain curves of the composites were recorded by a testing machine (Instron 3366, USA) according to the ASTM D412 standard at room temperature. The crosslinking densities of the composites were measured by the equilibrium swelling method at 25 °C [31]. The dielectric properties of the composites were determined by a dielectric analyzer (Concept 40) in the frequency range of 10^0^~10^4^ Hz under an electric voltage of 1 V at room temperature. Additionally, Cu electrodes were utilized during the dielectric testing. The dielectric samples consisted of films that were 20 mm in diameter and 0.3 mm in thickness. The dielectric properties of the samples were measured at room temperature, since DEA is usually used at room temperature in practical life, and ESO evaporates at high temperatures. The electromechanical properties of DE composite films were studied via a circular thin-film actuator, as according to our previous study [32]. During the actuation testing, the DE films would break when subjected to the highest electric field, even when considering dielectric strength. The historical dependence of the 50 phr ESO/Si747@BTO/ENR composite was measured under 15 kV/mm, until its breakdown.

## 3. Results and Discussion

### 3.1. Effect of Si747@BTO Nanoparticles on Actuation Performance of ENR Composites

In this study, Si747 was used to modify the surface of BTO nanoparticles and to improve the dispersion of BTO nanoparticles in the ENR matrix. The corresponding reaction mechanism can be described as follows: The Si747 was hydrolyzed in an alkaline buffer solution to generate silanol, which then reacted with hydroxyl groups on BTO nanoparticles to form covalent bonding via condensation reaction to yield Si747@BTO nanoparticles (Figure 1b). The presence of sulfhydryl functional groups allowed Si747 to participate in the vulcanization of the ENR chains (Figure 1c). This mechanism can be described as follows: First, the hydrogen ions lost from the sulfhydryl functional groups in Si747 generated thiol anions. Afterward, the thiol anions, with strong nucleophilicity, attacked the carbon atoms with low resistance on the epoxy functional groups to generate alkoxide anion intermediates, which, in turn, combined with hydrogen ions to produce thiol–epoxy reaction products [33].

**Figure 2:**
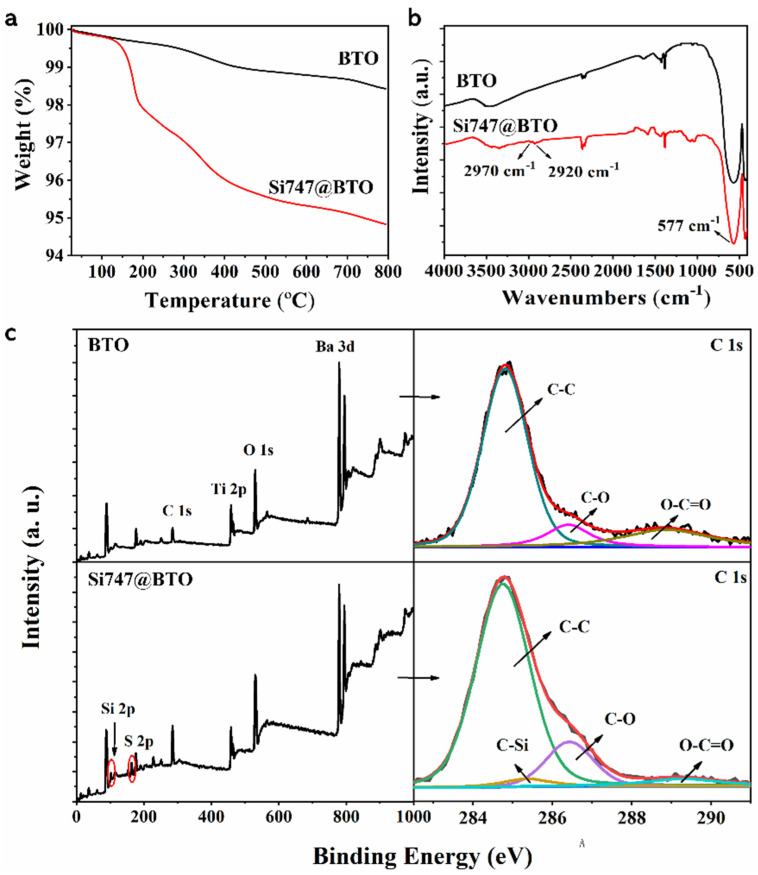
Structural analysis of BTO and Si747@BTO nanoparticles. (**a**) TGA curves, (**b**) FTIR spectra, and (**c**) XPS wide and C1s spectra.

The TGA curves of pristine BTO and Si747@BTO nanoparticles are compared in Figure 2a. Further, the mass residues of pristine BTO and Si747@BTO nanoparticles at 800 °C were determined to be 98.43% and 94.83%, respectively. The lower residual masses of Si747@BTO, when compared to the pristine BTO nanoparticles, can be attributed to the grafting of Si747. The FTIR spectra of pristine BTO and Si747@BTO nanoparticles are displayed in Figure 2b. The two new characteristic peaks at 2970 cm^−1^ and 2920 cm^−1^ were attributed to the -CH_2_ and -CH_3_ stretching vibration of Si747, respectively. The XPS wide-scan spectra, as well as the C1s spectra of BTO nanoparticles and Si747@BTO nanoparticles, are provided in Figure 2c. Compared to pristine BTO, the new Si 2p and S 2p peaks in Si747@BTO nanoparticles were attributed to the presence of Si747 (Figure 2c). In addition, the intensity of the peak corresponding to the O-C=O bond in the C1s spectrum was weakened in Si747@BTO nanoparticles when compared to BTO nanoparticles. The areas under the peaks of the C-C and C-O bonds of Si747@BTO nanoparticles also enlarged when compared to those of pristine BTO due to the presence of large numbers of ether bonds in Si747. The presence of C-Si bonds in the C1s spectrum of Si747@BTO nanoparticles confirmed the existing sulfhydryl groups in Si747. Overall, Si747 was successfully grafted to the surface of BTO nanoparticles.

**Figure 3:**
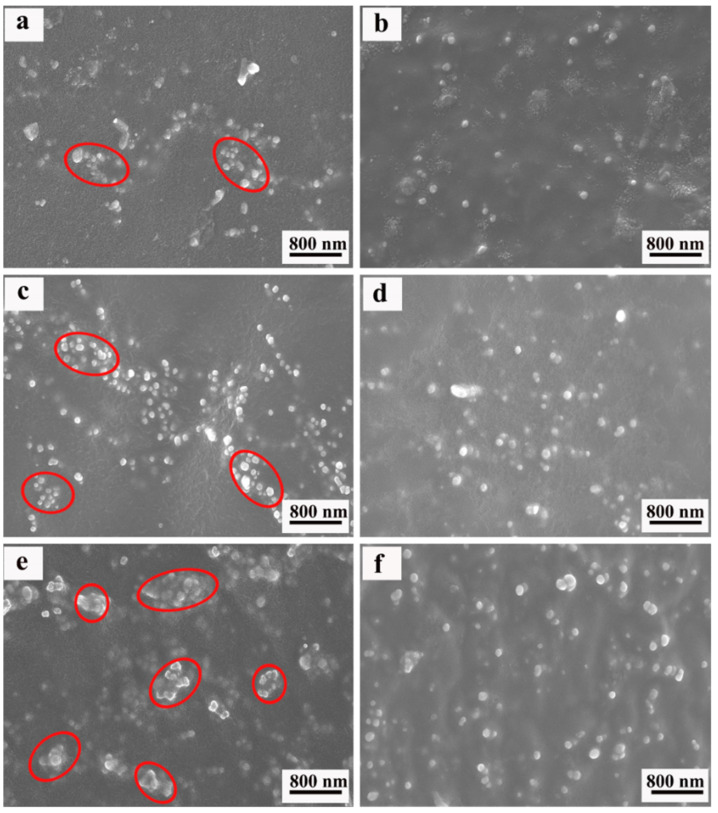
SEM micrographs of ENR composites. (**a**) 10 phr BTO/ENR, (**b**) 10 phr Si747@BTO/ENR, (**c**) 20 phr BTO/ENR, (**d**) 20 phr Si747@BTO/ENR, (**e**) 30 phr BTO/ENR, and (**f**) 30 phr Si747@BTO/ENR.

The SEM images of ENR composites, with various loadings of BTO and Si747@BTO nanoparticles, are provided in Figure 3. The red circle in SEM images represents the aggregation. Pristine BTO nanoparticles almost appeared to be exposed on the fracture surface of the ENR matrix (Figure 3a,c,e), indicating poor interfacial bonding between BTO nanoparticles and the ENR matrix. In addition, numerous BTO aggregations were appeared on the ENR matrix and increased with the content of BTO nanoparticles. However, the Si747@BTO nanoparticles were uniformly dispersed in the ENR matrix when compared to BTO/ENR composites (Figure 3b,d,f). Even after the increase in the content of Si747@BTO nanoparticles to 30 phr, small amounts of aggregations were found in the ENR matrix. In addition, the interface between Si747@BTO nanoparticles and the ENR matrix became more ambiguous than that of BTO/ENR composites. This might be due to the formation of strong covalent bonds between Si747 and ENR chains during the vulcanization process, thereby improving the dispersion and interfacial compatibility of polymeric composites [24].

**Figure 4:**
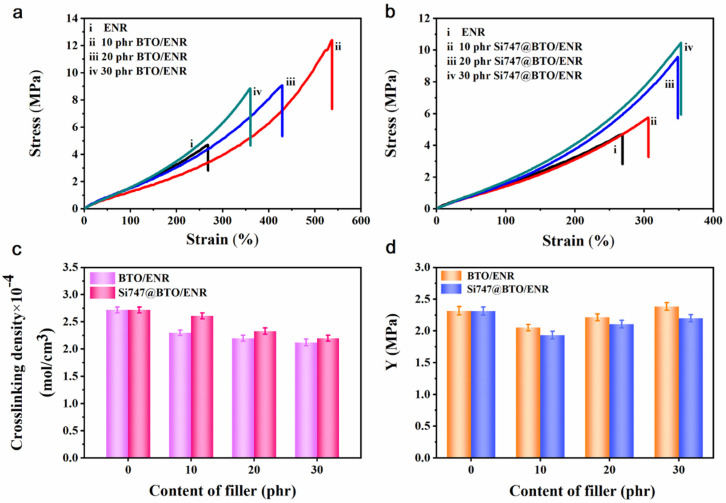
Typical stress–strain curves of ENR composites. (**a**) BTO/ENR and (**b**) Si747@BTO/ENR composites. (**c**) Crosslinking density, and (**d**) *Y* values of BTO/ENR and Si747@BTO/ENR composites.

The stress–strain curves of BTO/ENR and Si747@BTO/ENR composites are displayed in Figure 4a,b, respectively. The elongation at the break (*E_b_*) of ENR composites was larger than that of pure ENR. This was, therefore, ascribed to the decreased crosslinking density of ENR composites (Figure 4c). However, the *E_b_* value of Si747@BTO/ENR composites was inferior to that of BTO/ENR composites at the same filler content. The reason for this may have to do with the covalent bonds between Si747@BTO nanoparticles and the ENR chains restricting the slippage of molecular chains [24]. In addition, the tensile strength (*σ*) of ENR composites was also higher than that of pure ENR, which was attributed to the enhancement effect of BTO nanoparticles. As shown in Figure 4a,b, the Si747@BTO/ENR composites displayed higher *σ* values than those of BTO/ENR composites under the same filler content. For example, the stress of the 30 phr BTO/ENR composite reached 6.3 MPa at the strain of 300%, while that of the 30 phr Si747@BTO/ENR composite was 7.7 MPa. This can be explained by two main reasons. First, the BTO nanoparticles were evenly dispersed in the rubber matrix after modification by Si747, and the strengthening effect of the nanoparticles became more significant. Second, strong covalent bonds were existed between Si747@BTO nanoparticles and the ENR matrix, resulting in the uniform stress distribution of ENR composites [31,32].

As displayed in Figure 4d, the *Y* of the composites first decreased and then increased with filler content. The ENR composites filled with 10 phr pristine BTO and Si747@BTO nanoparticles both displayed the lowest *Y* value. In general, the *Y* of composites can be related to the competitive effect of crosslinking density and the filler network [34,35]. At 10 phr filler, the crosslinking density of the composite decreased and the filler network did not form in the rubber matrix (Figure 3b), leading to a decrease in the *Y* value. However, the filler network formed in the rubber matrix when nanoparticle loading increased to 20 phr and 30 phr, thereby clearly enhancing the *Y* with a weak effect of crosslinking density.

**Figure 5:**
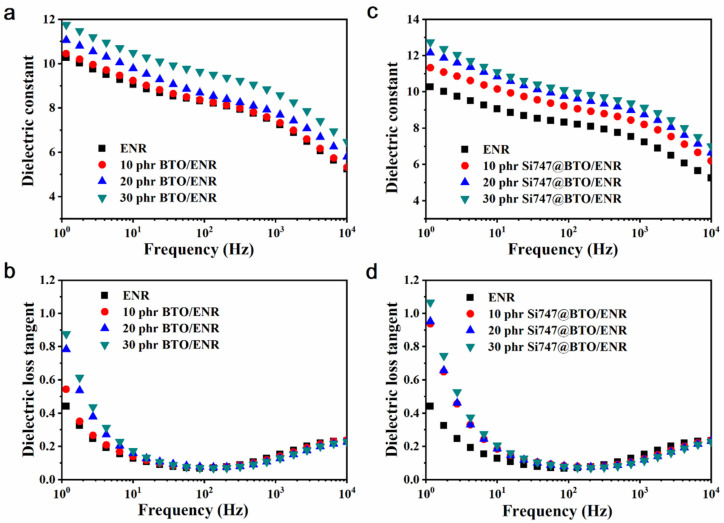
Frequency dependencies of *ε_r_* (**a**,**c**) and *tanδ* (**b**,**d**) in the ENR composites.

The changes in *ε_r_* and *tanδ* of BTO/ENR and Si747@BTO/ENR composites as a function of frequency are shown in Figure 5. The *ε_r_* of ENR composites decreased as the frequency increased due to the dipolar polarization of ENR chains (Figure 5a,c), as well as the interface polarization between the dielectric filler and the ENR matrix, which cannot keep up with the change in the electric field [36,37]. In addition, more doped BTO nanoparticles resulted in higher *ε_r_* values in the ENR composites. This can be attributed to BTO nanoparticles with higher *ε_r_* than that of the ENR matrix, leading to ENR composites with elevated *ε_r_*. Additionally, the introduction of interfacial polarization between the ENR matrix and the BTO nanoparticles enhanced the *ε_r_* of ENR composites. Moreover, the *ε_r_* of Si747@BTO/ENR composites was larger than that of BTO/ENR composites at the same filler content. For example, the 30 phr Si747@BTO/ENR composite showed an *ε_r_* of 10.06 at 100 Hz, a value higher than that of the 30 phr BTO/ENR composite (9.5 at 100 Hz). The increased *ε_r_* would be ascribed to the presence of Si747, which effectively enhanced the dispersion of BTO nanoparticles in the ENR matrix, resulting in stronger interfacial polarization [9,38]. The interface polarization not only increased the *ε_r_*, but also enhanced the *tanδ* of the ENR composites (Figure 5b,d) [39]. At a certain frequency, the Si747@BTO/ENR composites exhibited higher *tanδ* than that of the BTO/ENR composites, at the same filler content. However, the largest *tanδ* of the Si747@BTO/ENR composites was still less than 0.2 at 10 Hz, meaning that the electrostatic energy could efficiently be converted into mechanical energy [40].

**Figure 6:**
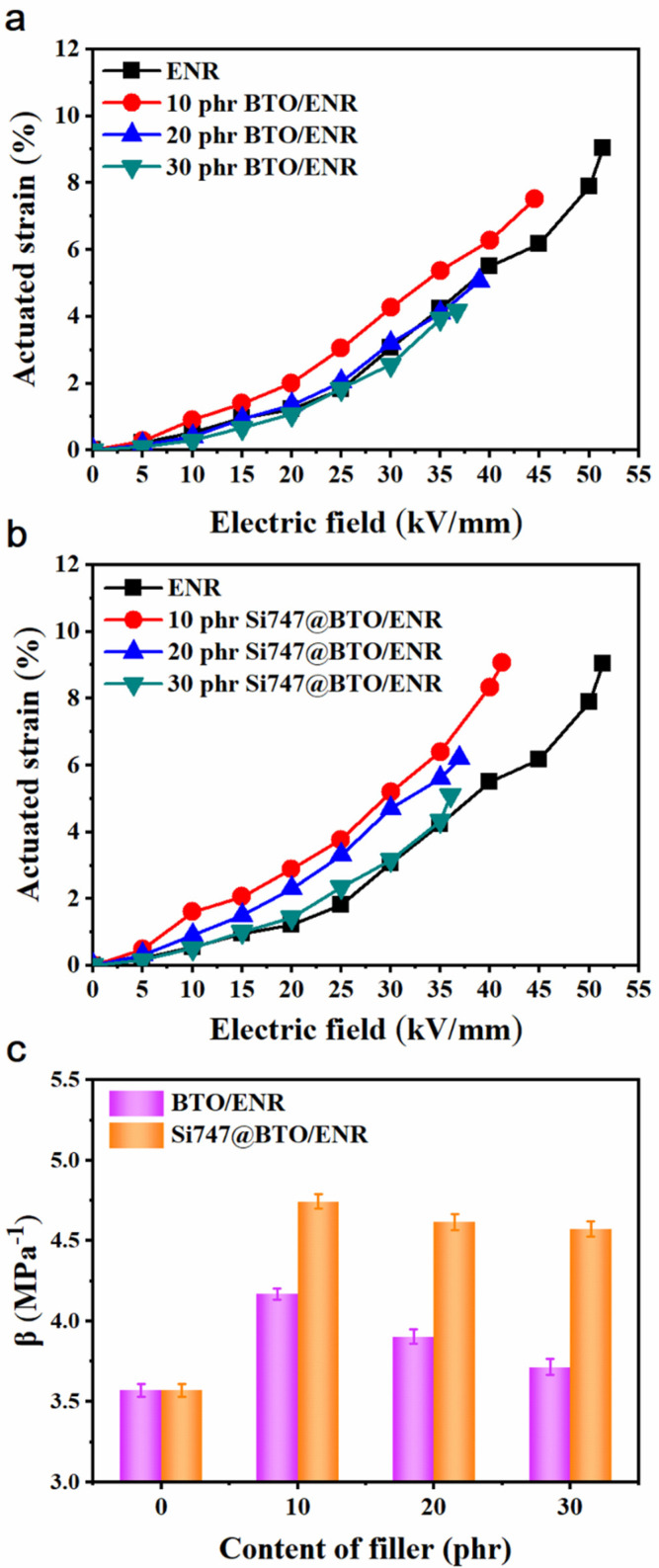
Actuated strains of ENR composites. (**a**) BTO/ENR, (**b**) Si747@BTO/ENR, and (**c**) *β* of ENR composites.

The changes in actuated strains of ENR composites with various loadings of BTO and Si747@BTO nanoparticles, as a function of the electric field, are displayed in Figure 6a,b, respectively. Under a certain electric field, BTO/ENR and Si747@BTO/ENR composites both showed the largest electrically actuated strains in the 10 phr filler. This can be interpreted by the lowest *Y* and increased *ε_r_* leading to the largest *β* (Figure 6c). In regard to nanoparticles from 20 phr and 30 phr, the actuated strains of ENR composites declined. However, the Si747@BTO/ENR composites displayed larger actuated strains than BTO/ENR composites at the same filler loading and electrical field. For example, the 10 phr BTO/ENR composite displayed an actuated strain of 6.27% at 40 kV/mm, while the 10 phr Si747@BTO/ENR composite showed an actuated strain of 8.34% within the same electric field. The improved actuated strains resulted from the larger *β* of Si747@BTO/ENR composites.

The dielectric strength of ENR composites also decreased with the increase in the filler amount. The gaps induced by the inorganic fillers and in the intervention of air, with a breakdown strength around 3 V/mm, led to reduced dielectric strength. After modification by Si747, the electric field intensity at the interface between dielectric nanoparticles and the ENR matrix became homogeneous, resulting in Si747@BTO/ENR composites with higher dielectric strengths than those of BTO/ENR composites [41,42].

### 3.2. Effects of ESO on Actuation Performance of ENR Composites

To further improve the actuation performance of Si747@BTO/ENR composites, a plasticizer of ESO was added to reduce its *Y* and improve *β*. The TGA and FTIR of ENR composites are provided in Figures S1 and S2, respectively. The mass residues of pure ENR, 10 phr Si747@BTO/ENR, and the 50 phr ESO/Si747@BTO/ENR composite at 800 °C, were determined as 4.9%, 12.4%, and 9.6%, respectively (Figure S1). The residual mass of the 50 phr ESO/Si747@BTO/ENR composite was lower than that of the 10 phr Si747@BTO/ENR composite, which was attributed to the evaporation of ESO. In Figure S2, the characteristic peak at 524 cm^−1^ in 10 phr Si747@BTO/ENR and 50 phr ESO/Si747@BTO/ENR composites was attributed to the stretching vibration of Ti-O bonds in BTO. However, compared to the 10 phr Si747@BTO/ENR composite, the new characteristic peak at 1743.2 cm^−1^ in the 50 phr ESO/Si747@BTO/ENR composite was assigned to the stretching vibration of C=O bonds in ESO [28].

**Figure 7:**
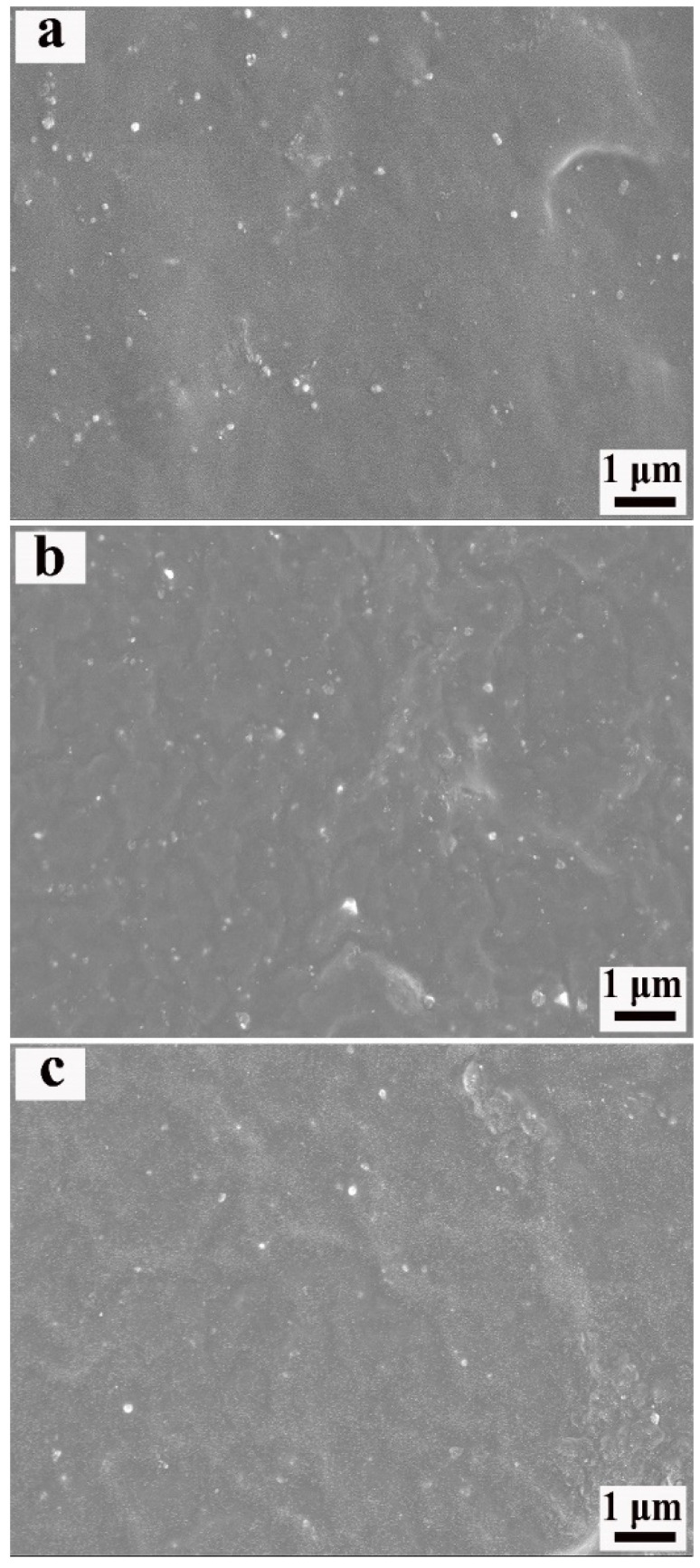
SEM images of the Si747@BTO/ENR composite with various loadings of ESO. (**a**) 10 phr, (**b**) 30 phr, and (**c**) 50 phr.

The microstructural images of Si747@BTO/ENR composites with various loadings of ESO are exhibited in Figure 7. The Si747@BTO nanoparticles looked uniformly dispersed in the ENR matrix with little aggregations after the addition of ESO. The number of Si747@BTO nanoparticles also decreased as the content of ESO increased. This can be explained by the small amount of effective content of Si747@BTO nanoparticles after the incorporation of ESO.

**Figure 8:**
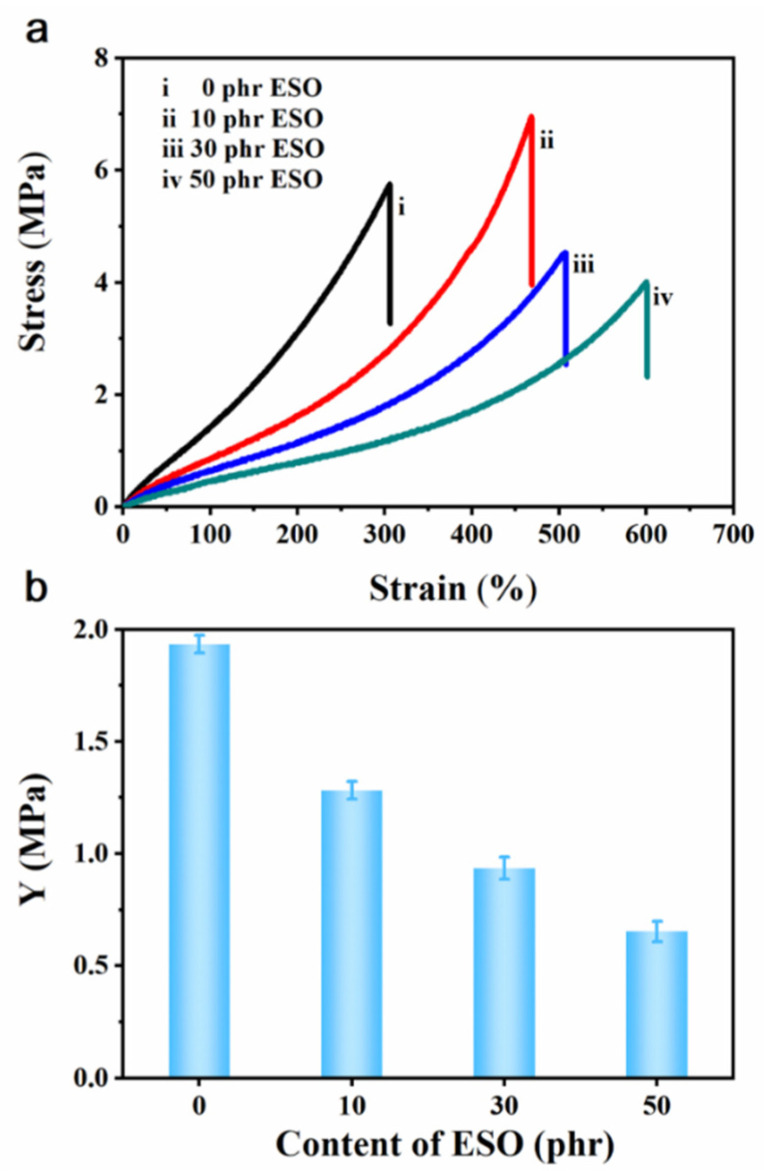
(**a**) Stress–strain curves of Si747@BTO/ENR composites with various loadings of ESO: (i) 0 phr, (ii) 10 phr, (iii) 30 phr, and (iv) 50 phr. (**b**) *Y* of 10 phr Si747@BTO/ENR composites with various loadings of ESO.

The stress–strain curves of ESO/Si747@BTO/ENR composites are summarized in Figure 8. The *σ* of ESO/Si747@BTO/ENR composites gradually decreased as the content of ESO rose (Figure 8a). The decreased *σ* was ascribed to the addition of ESO, which effectively decreased the volume fraction of BTO nanoparticles in the ENR matrix and destroyed the filler network in the ENR composites [17]. Meanwhile, the *E_b_* of ENR composites rose with the ESO content. The maximum elongation at the break of the 50 phr ESO/Si747@BTO/ENR composite reached 600%, while those of the 30 phr ESO/Si747@BTO/ENR and 10 phr ESO/Si747@BTO/ENR composites were 507% and 468%, respectively. This can be attributed to the ESO weakening the interactions between the ENR molecular chains, thereby enhancing the motility of macromolecular chains. Simultaneously, the ESO, as a small molecule, increased the distance between ENR chains, thereby sharply reducing the *Y* of the ENR composites [43]. In Figure 8b, the *Y* of the ENR composite filled with 50 phr ESO reduced to 0.65 MPa, thereby inducing larger actuated stains at low electrical fields.

**Figure 9:**
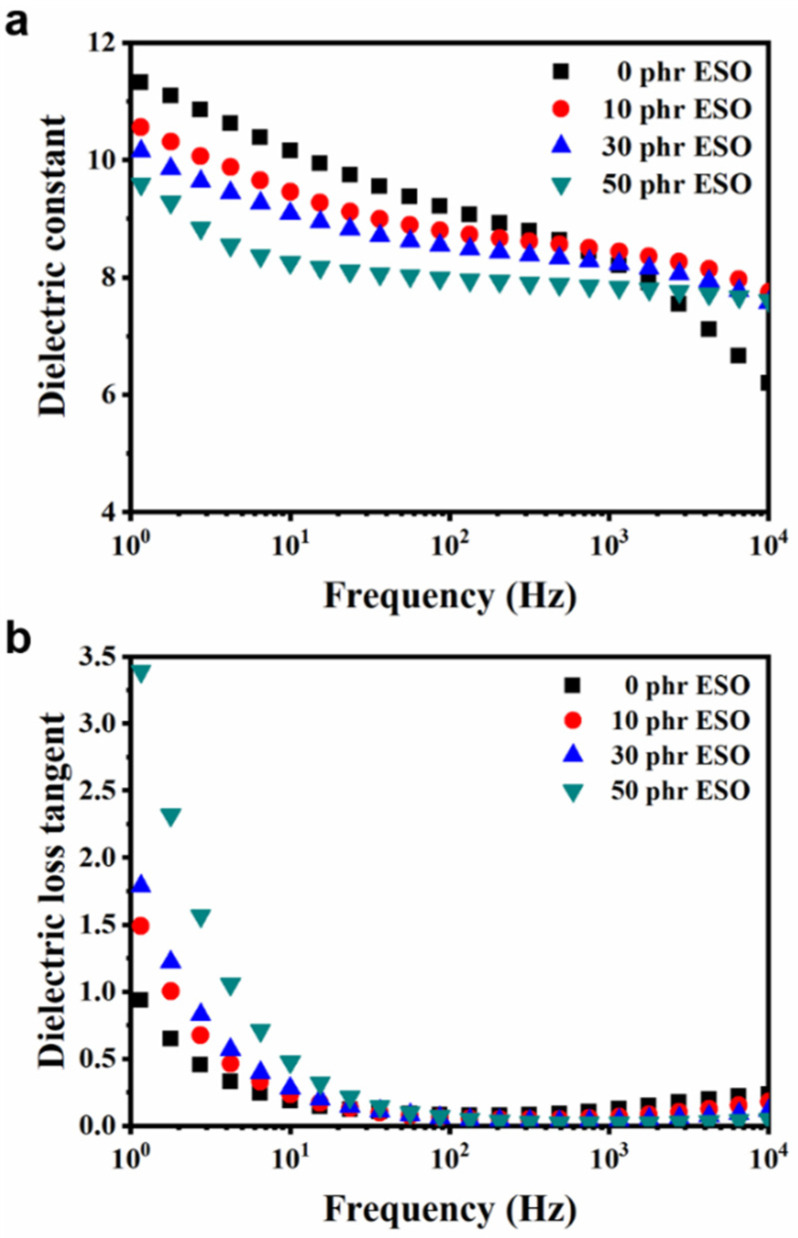
Frequency dependence of (**a**) *ε_r_* and (**b**) *tanδ* of Si747@BTO/ENR composites with various loadings of ESO.

The dielectric behaviors of Si747@BTO/ENR composites with various loadings of ESO are displayed in Figure 9. The *ε_r_* decreased with the increase in content of ESO (Figure 9a), due to the lower *ε_r_* of ESO (7.8 at 1 kHz) than that of the 10 phr Si747@BTO/ENR composites (8.3 at 1 kHz). The decreased effective volume concentration of Si747@BTO nanoparticles in the ENR matrix also weakened the interfacial polarization. Meanwhile, *ε_r_* declined as frequency rose, due to the polarization of the ENR composites that cannot keep pace with the change in frequency [44]. As shown in Figure 9b, the ESO increased the *tanδ* of Si747@BTO/ENR composites even though it decreased the *ε_r_* of the ENR composites. The increased *tanδ* may be attributed to the liquid characteristics of ESO [45]. However, the largest *tanδ* was lower than 0.47 at 10 Hz, meaning that small amounts of energy were lost via dielectric loss during actuation.

**Figure 10:**
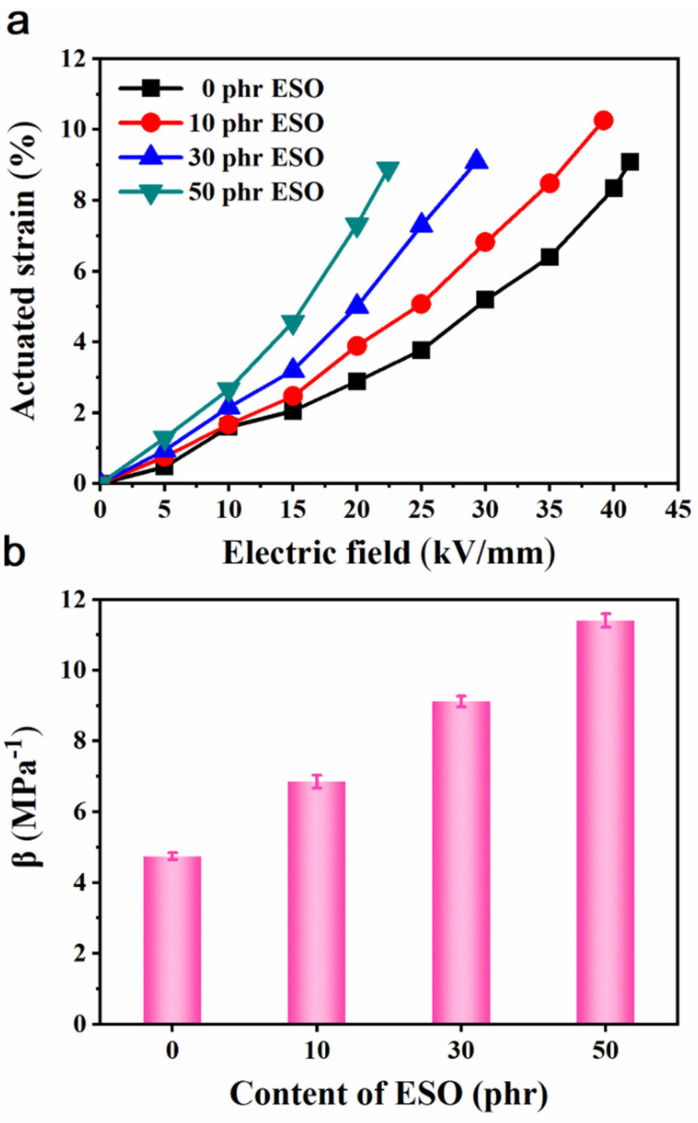
(**a**) Actuated strains and (**b**) *β* of Si747@BTO/ENR composites with various loadings of ESO.

The actuated strains of Si747@BTO/ENR composites, which are filled with different contents of ESO, are illustrated in Figure 10a. The actuated strains of the ESO/Si747@BTO/ENR composites increased sharply as a function of ESO content. The Si747@BTO/ENR composite filled with 50 phr ESO showed a maximum actuated strain of 8.89% at 22 kV/mm. This value was about 5.1-fold higher than that of pure ENR (1.45%) under the same electric field. Some similar works were compared in Table 1, where ESO/Si747@BTO/ENR composites displayed a relatively enhanced actuated strain. The enhanced actuated strain was ascribed to ESO, which not only destroyed the filler network in the ENR composite, but also weakened the molecular chain entanglement, thereby leading to lower *Y* and improved *β* (Figure 10b). In Figure 10b, the *β* increased to 11.4 MPa^−1^, while that of pure ENR was only 3.5 MPa^−1^. Furthermore, the dielectric strength of ESO/Si747@BTO/ENR composites declined with ESO content due to the increment in the formed defects by ESO [46]. However, the dielectric strengths of all ESO/Si747@BTO/ENR composites were larger than 22 kV/mm, thereby judging a relatively safe electrical field.

**Figure 11:**
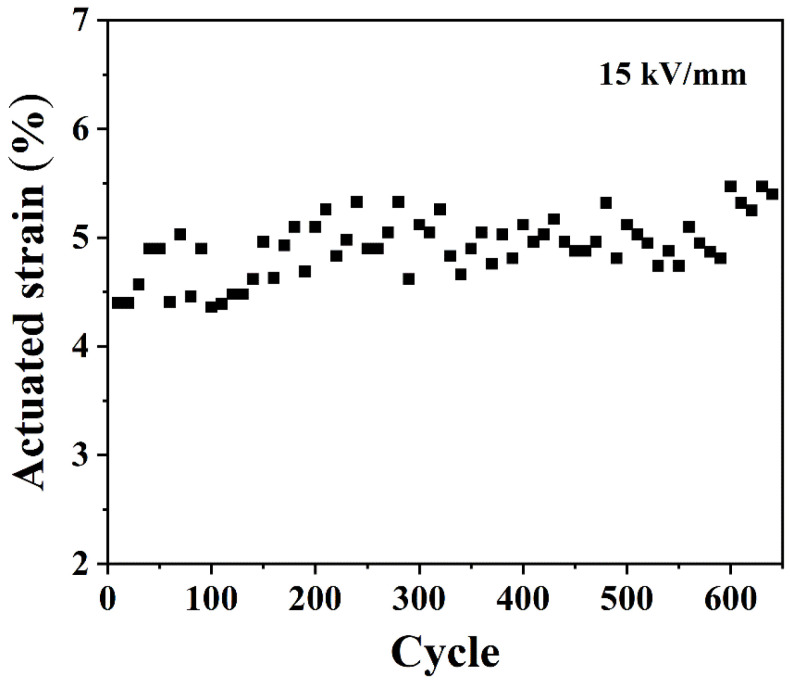
The actuated strain of 50 phr ESO/Si747@BTO/ENR DEA under a cyclic electric field of 15 kV/mm.

The history dependence of 50 phr ESO/Si747@BTO/ENR DEA under a cyclic electric field of 15 kV/mm is provided in Figure 11. The maximum cycle for the 50 phr ESO/Si747@BTO/ENR composite reached 640. In addition, the 50 phr ESO/Si747@BTO/ENR DEA displayed relatively stable actuated strain with a little increment during cycling. The slightly increased actuated strain might be ascribed to the creep processes of ENR chains [47].

**Table 1:** Values of the actuation performance of different DEs, without pre-strain, reported in previous works and the present work.

Samples	Area-Based Actuated Strain (%)	Electrical Breakdown Field (kV/mm)	Enhancement of Actuated Strain (Fold)	Reference
20 phr TiO2/PDA/KH570/NBR	16	60	0.5	[31]
16 vol% TiO2@SiO2/PDMS	6.08	30 V/μm	1.8	[48]
5 wt% PDA@SiO2@rGO/PDMS	14.23	33.19	4.7	[40]
50 phr BT/PDA/HNBR	20	45	0.54	[49]
10 wt% BT/PVDF/HNBR	10	100	1 *	[50]
2.0 wt% TGNPs/PDMS	3.6	15	1.6	[51]
10 wt% WI/10 wt% HNT/MVSR	16.22	59.31	1.7	[52]
10 wt% wp-TiO2/SEBS	21.5	34 V/μm	1.9 *	[53]
5 wt% CCTO/PDMS	33.8	14	0.7 *	[54]
15 vol% PDVB@PANI/PDMS	10	50 V/μm	1.1	[55]
0.5 phr GNS-PDA/XNBR	2.4	18	0.9	[56]
100 phr DMSO/TiO2/PDMS	13	30 V/μm	2.3	[57]
30 wt% ESO/10 wt% TiO2/HNBR	13.6	30	1.3	[58]
0.5 vol% rGO-300/ PDMS	13.32	20	1.6	[59]
3 vol% Gr /CNT/TPU	2	7.5	3.4	[60]
0.5 vol% RGO/CNS/XNBR	5.68	7	1.1	[61]
50 phr ESO/Si747@BTO/ENR	8.89	22	5.1	This work

The numbers with * are estimated from figures.

## 4. Conclusions

The covalent-bond-functionalized Si747@BTO nanoparticles and ESO plasticizer were simultaneously incorporated into the ENR matrix to prepare high-performance DEAs. The participation of Si747 present on surfaces of BTO nanoparticles, during the vulcanization of ENR chains, led to improved interfacial interaction between the BTO nanoparticles and the ENR matrix, thereby enhancing the *ε_r_*. Furthermore, the ESO destroyed the filler network and weakened the interaction between polymer chains, sharply reducing *Y* and significantly increasing *β*. An actuated strain of 8.89% was achieved by the 50 phr ESO/Si747@BTO/ENR composite at 22 kV/mm, a value 5.1-fold higher than that of pure ENR (1.45%) under the same electric field. Moreover, the ENR DEA exhibited good stability with a slight increment during cycling actuation, demonstrating its potential in practical applications.

## Supplementary Materials

The following supporting information can be downloaded at: https://www.mdpi.com/article/10.3390/polym14194218/s1, Figure S1: TGA curves of pure ENR, 10 phr Si747@BTO/ENR, and 50 phr ESO/ Si747@BTO/ENR composite. Figure S2: FTIR spectra of pure ENR, 10 phr Si747@BTO/ENR, and 50 phr ESO/Si747@BTO/ENR composite.
