# Supplementary material for: Enhanced Actuation Performance of Polymeric Composites by Simultaneously Incorporating Covalent-Bond-Functionalized Dielectric Nanoparticles and Polar Plasticizer
Source: Polymers (Basel). 2022 Oct 8;14(19):4218. doi: 10.3390/polym14194218 (PMC9572819; doi:10.3390/polym14194218)
Supplement: Supplementary file 1 [file polymers-14-04218-s001.zip › polymers-1893102-supplementary.pdf]

Supplementary Information

Enhanced Actuation Performance of Polymeric Composites by Simultaneously Incorporating Covalent-Bond-Functionalized Dielectric Nanoparticles and Polar Plasticizer

Huiwan Lu,[1,2] Dan Yang[1,*]

[1]College of Materials Science and Engineering, Beijing University of Chemical Technology, Beijing University of Chemical Technology, Beijing 100029, China

[2]College of New Materials and Chemical Engineering, Beijing Institute of Petrochemical Technology, Beijing 102617, China

[*]Corresponding author. E-mail: D. Yang (danyang@buct.edu.cn)

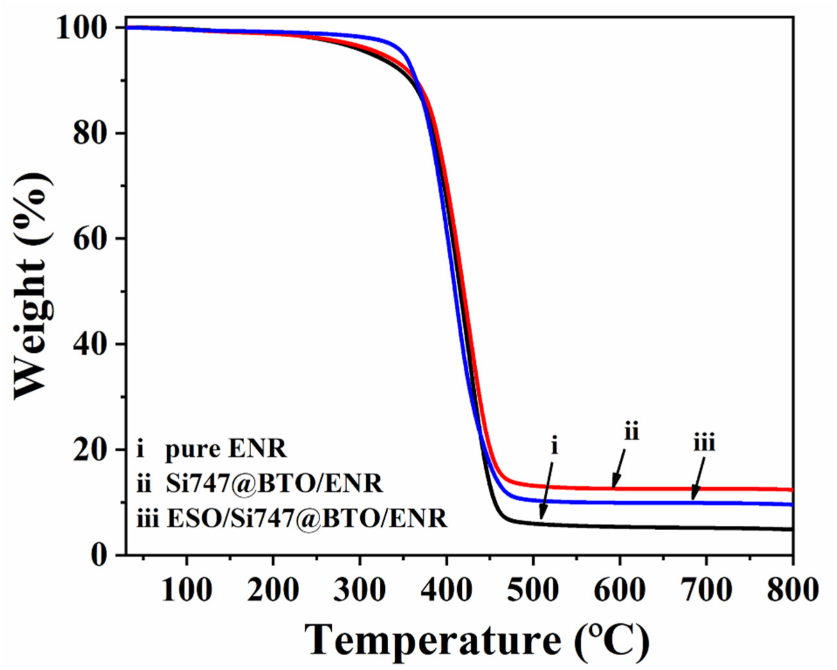

Figure S1. TGA curves of pure ENR, 10 phr Si747@BTO/ENR, and 50 phr ESO/Si747@BTO/ENR composite.

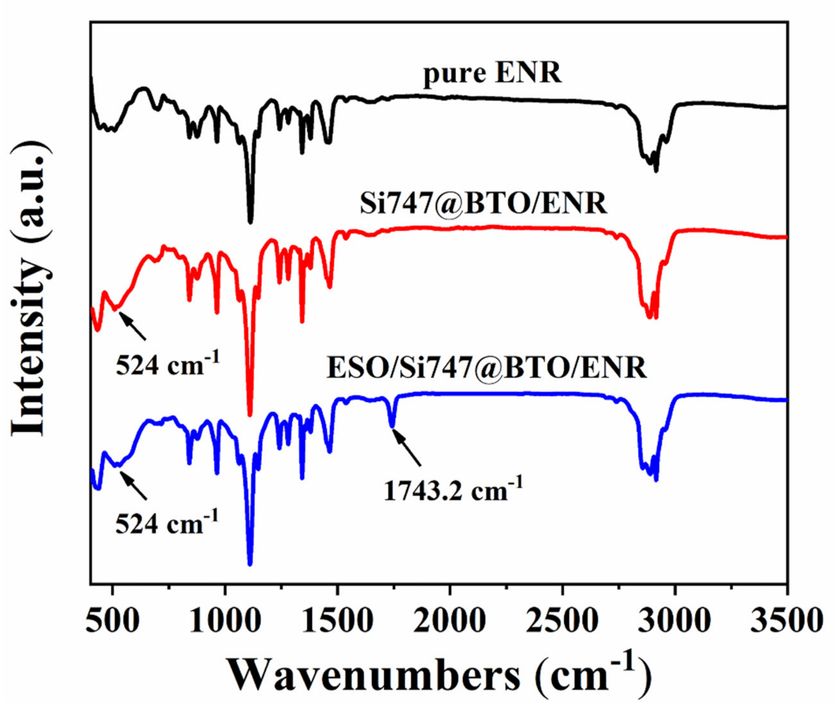

Figure S2. FTIR spectra of pure ENR, 10 phr Si747@BTO/ENR, and 50 phr ESO/Si747@BTO/ENR composite.
